# Advances in Electrochemical Nanobiosensors for Noninvasive Stress Biomarker Detection

**DOI:** 10.3390/s25237255

**Published:** 2025-11-28

**Authors:** Leonardo Messina, Paolo Amadoro, Maria Teresa Giardi

**Affiliations:** 1 Microsis srl, Via degli Olmetti, 8a, 00060 Formello, Italy; 2 Biosensor srl, Via degli Olmetti 44, 00060 Formello, Italy; p.amadoro@biosensor.it (P.A.); mt.giardi@biosensor.it (M.T.G.)

**Keywords:** cortisol detection, wearable biosensors, blood/sweat correlation, electrochemical biosensors

## Abstract

Cortisol is a key stress-related hormone whose accurate monitoring is essential for assessing physiological responses. Traditional detection methods are invasive and impractical for continuous use. This mini-review highlights recent advances in electrochemical biosensors, particularly immunosensors, aptasensors, and molecularly imprinted polymers for non-invasive cortisol detection in blood, saliva, interstitial fluid, and sweat. While saliva correlates strongly with serum free cortisol and is widely used in psychoneuroendocrinology, its reliance on active user compliance makes it unsuitable for continuous monitoring. In contrast, interstitial fluid and sweat offer routes for minimally invasive or fully non-invasive, on-body sampling, with sweat emerging as particularly promising due to its accessibility and correlation with serum analysis, paving the way for future personalized health applications. In this mini-review, we provide a concise overview of electrochemical biosensors for cortisol in blood, saliva, and interstitial fluid, and place particular emphasis on wearable sweat-based platforms, using the former matrices mainly as reference points for performance comparison and physiological validation.

## 1. Introduction

In circumstances where the human body is subjected to stress, a multitude of physiological processes are initiated with the objective of restoring the body to its normal state. Cortisol is a direct product of these physiological processes. It is produced by the adrenal fasciculata cells in response to the adrenocorticotropic hormone (ACTH) [1]. Cortisol is frequently designated the ‘stress hormone’ because its production is known to increase under conditions of severe psycho-physical stress. For instance, this may occur after extremely intense and prolonged physical exercise [2] or surgery [3]. It has been shown that its levels increase in case of prolonged fasting and incorrect eating habits, in fact skipping breakfast or consuming excessive quantities of food in a single daily meal can create a raise in the latter [4]. This hormone inhibits nonessential bodily functions in the short term to ensure maximum support for vital organs. It has been shown to induce an increase in cardiac output, resulting in raised blood sugar levels. This translates into increased hepatic gluconeogenesis, the process of converting alanine into glucose. This process also reduces the immune system’s defenses, stimulating glucagon secretion and decreasing insulin receptor activity. All these processes, in turn, have been shown to reduce inflammatory reactions and inhibit phospholipase A2 [5] thus leading to a decrease in collagen synthesis and bone matrix formation, with consequent acceleration of the onset of osteoporosis. While this process does favor the mobilization and use of fatty acids, in certain areas it has been observed to stimulate lipogenesis [6].

**Figure 1:**
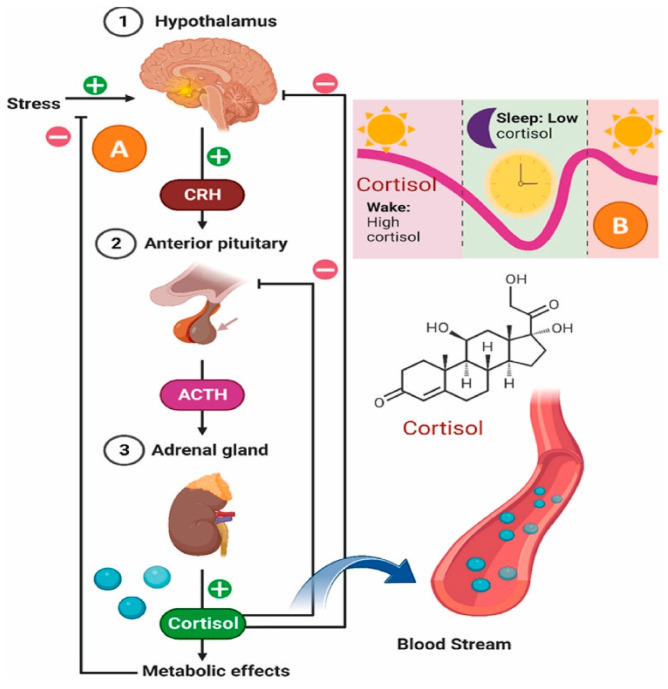
Diagram of the HPA (Hypothalamic–pituitary–adrenal) axis, illustrating its role in coordinating stress adaptation via feedback mechanisms and daily cortisol rhythms. Reprinted with permission from [10]. Copyright 2025 Elsevier.

It is important to examine the relationship between the endocrinal pathway for stress regulation to facilitate a comprehensive understanding of the physiological effects and the extent of damage inflicted during periods of exposure to stress (Figure 1). The hypothalamic–pituitary–adrenal (HPA) axis is the main endocrine pathway regulating the stress response via allostasis. In response to stress, the hypothalamus releases corticotropin-releasing hormone (CRH), which stimulates the pituitary gland to secrete ACTH into the bloodstream. ACTH then acts on the adrenal cortex, inducing steroidogenesis and the production of glucocorticoids, particularly cortisol [7]. Cortisol helps maintain physiological balance by exerting potent anti-inflammatory effects. It can induce apoptosis in T lymphocytes, preventing immune overactivation, and it strengthens endothelial tight junctions, limiting immune cell entry across the blood–brain barrier. Most importantly, cortisol binds to the glucocorticoid receptor (GR) and suppresses transcription of pro-inflammatory cytokine genes, such as TNF-α, leading to a broad downregulation of the immune response [8,9].

In recent years, research on stress biomarkers such as cortisol has increasingly focused on the development of minimally invasive monitoring systems. Unlike blood-based assays, wearable biosensors enable real-time hormone tracking through alternative biofluids, including saliva, interstitial fluid, and sweat. These approaches aim to overcome the discomfort and logistical constraints of conventional sampling methods. Several comprehensive reviews have examined these methodologies, analyzing their working principles, material strategies, and current technological progress. For instance, Duan et al. [10] investigated the applications of biosensors, emphasizing the detection of a variety of biochemical markers, including small molecules, hormones, drugs, and macromolecules, in biofluids such as interstitial fluid (ISF), sweat, wound exudate, saliva, and tears. The study by Karuppaiah et al. [11] is noteworthy for its comprehensive examination of the advancements made in the field of electrochemical sensors for cortisol detection. The study encompasses the design of these sensors, the underlying principles, and the electroanalytical methodologies employed. It further discusses potential strategies for electrode design and provides illustrative examples of sensing approaches. Karachaliou et al. [12] provided a comprehensive overview of the majority of the reported cortisol immunosensors, with a particular emphasis on electrochemical and optical sensors. The study delved into the underlying immunosensing detection principles of these sensors. Unlike previous studies, this mini-review exclusively focuses on a detailed analysis of recent research on electrochemical biosensors based on antibody and aptamer applications, emphasizing new nanomaterials, and on cortisol as a single stress biomarker across multiple biofluids (blood, interstitial fluid, saliva and sweat), with particular emphasis on non-invasive and wearable electrochemical platforms. In contrast to previous broad overviews of wearable electrochemical biosensors and cortisol detection strategies, we organize the discussion by biological matrix and then by recognition strategy (immunosensors, aptasensors and molecularly imprinted polymers), providing a unified framework that highlights how physiological cortisol levels, sample composition and lag times between compartments dictate sensor design and transduction choices. Several material families recur across recent devices for boosting charge transfer, immobilization density, antifouling and mechanical compliance: these include MXenes and MXene–MWCNT (multiwalled carbon nanotube) hybrids (high conductivity and surface area); metal–organic framework (MOF) nanoarchitectures (porosity and ordered bioreceptor orientation); laser-induced graphene (LIG) and graphene oxide (GO–COOH) (large electroactive area and easy functionalization); gold nanoparticles and AuNP-decorated or MWCNT-modified gold (signal amplification); Prussian blue (PB)-embedded molecularly imprinted polymer (MIP) films (built-in redox transduction); mesoporous silica on indium-tin oxide (ITO); and conducting hydrogels such as Poly(3,4-ethylenedioxythiophene) PEDOT/alginate or Polyaniline PANI (antifouling and soft contact). We review how these materials couple to antibodies, aptamers and MIPs/electrochemical MIPs (EC-MIPs), with a specific emphasis on sweat-based wearable devices, where label-free or internally referenced readouts are generally favored. Within this framework, blood, saliva and interstitial fluid (ISF) are discussed more concisely: they serve primarily to define physiologically relevant cortisol ranges, illustrate representative electrochemical sensing strategies, and provide reference points for matrix-specific challenges. The core objective of this mini-review is therefore to critically appraise recent advances in electrochemical nanobiosensors for sweat cortisol monitoring, while maintaining data comparability across matrices by systematically reporting performance metrics and physiological concentration ranges for blood, saliva and ISF. A focused literature analysis was performed to identify the main research gaps in cortisol biosensing. The results highlight that, while numerous studies report promising technical advances, few address the translation of electrochemical biosensors into real-world healthcare applications. To bridge this gap, the present review selectively examines the most recent and innovative works employing advanced nanomaterials for electrochemical cortisol detection. In addition, a concise market overview was carried out to assess the current level of commercialization of cortisol biosensing technologies. This analysis revealed a clear gap between laboratory prototypes and deployable wearable systems, underscoring the need for further integration of materials science, electronics, and data analytics to achieve clinically viable solutions and to translate cortisol biosensors into on-body, real-time monitoring devices.

Building on our earlier work on lactate biosensing [13], the present review shifts the focus toward cortisol as a representative stress biomarker, addressing distinct biochemical and electrochemical challenges associated with its detection. Within this framework, the EU-funded H2TRAIN initiative aims to bridge the gap between laboratory research and wearable healthcare technologies. The project follows an integrated strategy that spans sensor design, microfabrication, and electronic coupling with smart textiles and AI-driven data interpretation. Validation activities will take place across medical and sports applications, guided by social and ethical considerations to ensure user-centered development. Through the direct involvement of end users and multidisciplinary partners, H2TRAIN seeks to deliver next-generation wearable biosensing systems that combine technological innovation with practical clinical relevance [14,15].

Against this multidisciplinary background, the following sections examine how electrochemical biosensors operate across different biological matrices, outlining the specific analytical requirements and material strategies that enable reliable cortisol detection.

## 2. Biological Matrices and Biosensors

Monitoring cortisol levels in biological fluids provides essential information about an individual’s health and stress status, reflecting both endocrine and metabolic balance. Conventional analytical approaches, such as blood or saliva testing, are often invasive, time-consuming, and dependent on specialized personnel and laboratory facilities, making them unsuitable for continuous or real-time assessment. Recent advances in biosensor technology are transforming this scenario by enabling direct, noninvasive detection of cortisol with high sensitivity and temporal resolution. These systems open new possibilities for tracking circadian variations in the hormone, offering a more accurate and personalized view of physiological stress. To ensure measurement reliability, however, several critical aspects must be optimized: the selection of appropriate transducer materials, the choice of recognition elements capable of specific cortisol binding, and the electrochemical technique used for signal acquisition. Careful control of these parameters is fundamental to designing biosensors that perform consistently across diverse biofluids and real-world conditions. Material constraints differ by matrix. For example, blood favors PEDOT:PSS inks for small-volume, rapid Electrochemical Impedance Spectroscopy EIS at physiological levels. Interstitial Fluid ISF platforms use Au/3,3′-Dithiodipropionic acid di(N-hydroxysuccinimide ester) DTSP microarrays for oriented antibodies and AuNP-enhanced microneedles to enable real-time transdermal readout. Sweat wearables prioritize compliant, porous and anti-fouling interfaces, such as graphene/LIG, MXene-based composites, MOFs, GO–COOH, polystyrene (PS)-embedded MIPs, PEDOT/alginate hydrogels, indium tin oxide (ITO)/mesoporous silica nanoparticles (MSN) and PANI, to enable robust label-free or internally referenced sensing during motion and variable pH/ionic strength. The analysis of cortisol can be facilitated by immunosensors, which are capable of acting using specific antibodies linked to the surface of the substrate via cross-linkers. These cross-linkers guarantee very strong interactions with the immunogen. Another innovative method that is gaining ground in the analysis of cortisol is the one that includes the use of aptamers, which are nucleic acids capable of binding to a molecule or a protein. Consequently, these cells undergo a structural conformational variation, thereby producing physical signals. These signals are then translated into electrical signals by a transducer. Another type of electrochemical biosensor that has been employed extensively involves MIPs, which are polymers capable of selectively recognizing the analyte and which are a superior alternative to immunosensors.

As cortisol detection can be performed in different biological matrices, this review first provides a concise overview of electrochemical biosensors in blood, saliva and interstitial fluid, followed by a more detailed analysis of sweat-based platforms. This structure is intended to maintain data comparability between matrices (in terms of physiological ranges, sensing strategies and analytical performance), while reflecting the primary focus of the article on wearable sweat cortisol monitoring.

### 2.1. Blood Analysis

**Figure 2:**
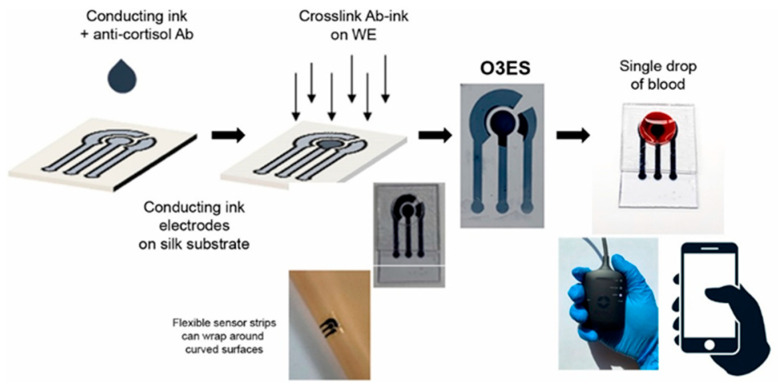
Schematic overview of device fabrication and measurement process, highlighting compatibility with a portable handheld unit and dual use in rigid and flexible modes (inset). Reprinted with permission from [22]. Copyright 2025 Elsevier.

Blood testing remains the gold standard for evaluating physiological status, yet its invasive nature can trigger stress responses, temporarily elevating biomarkers such as cortisol. In circulation, about 80–90% of cortisol is bound to corticosteroid-binding globulin (CBG) and 10–15% to serum albumin, leaving only 5–10% in its free, biologically active form. This unbound fraction is responsible for regulating stress-related physiological functions. Therefore, focusing on the detection of free cortisol, preferably through noninvasive biofluids such as sweat or saliva, can provide a more accurate and real-time assessment of stress levels than conventional blood analysis. [16]. The nominal value of cortisol in the blood, which has a half-life of 80 min [17], also varies depending on the circadian cycle, with maximum values recorded during the morning and minimum values recorded during the night [18]. Its concentration range is thus approximately 20–250 ng/mL [19]. The active molecular fraction of cortisol, termed the Cortisol Free Index (CFI), is typically detected through the application of the Coolen equation, which is based on the complete measurement of the biomolecule in its active and inactive states [20]. To achieve this objective, a range of commercially available cortisol measurement techniques are employed, including high-performance liquid chromatography (HPLC), enzyme-linked immunosorbent assay (ELISA), chemiluminescence immunoassay (CLIA), and surface plasmon resonance (SPR). Nevertheless, these approaches are now considered to be outdated due to the numerous critical issues that can arise during their utilization. Although blood sampling has long been employed as a standard approach for physiological and biochemical analysis, it is inherently invasive and often causes discomfort or anxiety in subjects. The procedure also carries a risk of infection, particularly with repeated sampling or in vulnerable individuals. Moreover, several studies have reported that the act of blood collection itself can induce transient increases in circulating cortisol levels, potentially altering the very biomarker being measured [17]. This paradox highlights a key limitation of traditional blood-based assays for assessing stress-related hormones, emphasizing the need for alternative, noninvasive sampling methods, such as those based on sweat, saliva, or interstitial fluid, that minimize stress-induced bias and provide a more accurate reflection of true physiological conditions. Furthermore, cortisol, when present in blood at ambient temperature, is inherently unstable. Consequently, it necessitates heightened safety measures by highly skilled personnel, along with a concomitant increase in handling expenses. Following the sampling stage, further laborious techniques must be employed, including ultrafiltration, equilibrium dialysis and steady state gel filtration [21]. An alternative to this type of procedure could be the use of real-time or point-of-care (PoC) applications, such as electrochemical biosensors. The latter can bypass all the aforementioned problems. Pradhan et al. [22] developed a disposable PEDOT:PSS-based electrochemical immunosensor designed for rapid cortisol detection from a minimal blood sample (Figure 2). By embedding anti-cortisol antibodies directly within the conductive polymer matrix, the authors achieved direct, label-free electrochemical impedance measurements without the need for external reagents or pretreatment. The device demonstrated high sensitivity across physiological ranges and operated effectively with microliter-scale volumes, underscoring its suitability for portable and point-of-care diagnostics. This work illustrates the potential of conductive polymer platforms for creating sustainable, low-cost biosensors capable of real-time hormone monitoring in complex biological fluids.

Nevertheless, for the purpose of measuring cortisol, blood has been collected in recent years for this application. In lieu of them, biological matrices such as sweat and ISF are favored, as they have the capacity to render cortisol detection protocols less invasive. Consequently, this ensures an enhancement in terms of both handling cost and patient well-being.

### 2.2. Interstitial Fluid Analysis

The analysis of biological fluids such as blood and saliva plays a crucial role in monitoring cortisol levels and understanding stress-related physiological responses. However, blood collection is inherently invasive and therefore unsuitable for continuous or frequent monitoring. Similarly, saliva sampling, though noninvasive, depends heavily on patient compliance and may be influenced by factors such as hydration status and circadian variability. In contrast, interstitial fluid (ISF) represents a highly promising alternative matrix for cortisol detection. ISF reflects the biochemical composition of plasma while being accessible through minimally invasive techniques that allow continuous collection directly from the skin surface, for instance via microneedles or gentle transdermal extraction. This makes ISF an ideal medium for real-time hormonal monitoring, bridging the gap between clinical accuracy and user comfort, and opening new possibilities for wearable biosensing technologies aimed at personalized health tracking.

**Figure 3:**
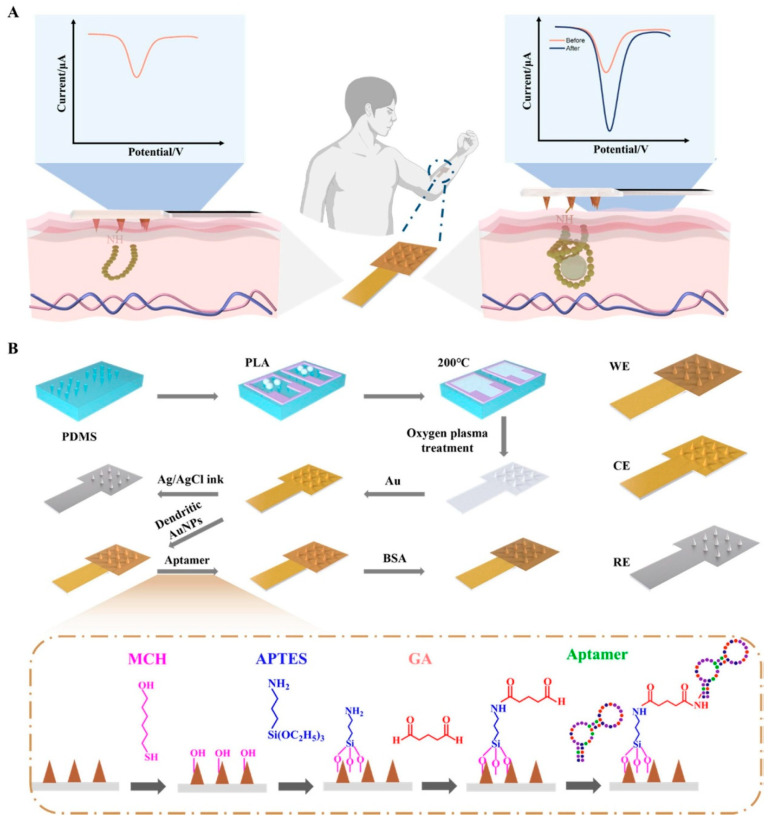
(**A**) Operating principle of the microneedle (MN) electrochemical biosensor; (**B**) Schematic of the fabrication steps (WE: working electrode; CE: counter electrode; RE: reference electrode). Reprinted with permission from [30]. Copyright 2025 Elsevier.

ISF is an extracellular fluid that surrounds the cells of the human body. It is composed of small and medium-sized molecules, including glucose, ethanol and cortisol itself. The collection of interstitial fluid is a technique that, while minimally invasive, does not require patient compliance. A simple ISF harvesting device can be utilized to harvest ISF continuously. These harvesting devices have been shown to be capable of harvesting ISF over a period of 2–3 days without the need for additional patient compliance [23,24]. The site of ISF extraction can be selected with a high degree of precision, with the procedure being carried out in almost any location on the human body (e.g., arms, abdomen, legs) without any loss of accuracy. The temporal discrepancy between blood and ISF levels has the potential to induce measurement errors in continuous monitoring systems. As demonstrated by Stout et al. [25], the application of modulated pressure resulted in a mitigation of the ISF physiological error by an average of 95%. Consequently, it is evident that periodic calibration of the sensor using blood samples to obtain metabolite concentrations in the ISF is not a prerequisite [26,27]. The most widely utilized electrochemical technique for the detection of cortisol in interstitial fluid is EIS. For instance, in the work conducted by [23], a minimally invasive technique was employed for the continuous extraction of interstitial fluid (ISF) from the stratum corneum (SC) [US Patent 6,183,434; Altea MicroPor™ Laser]. The method utilizes a low-energy near-infrared laser applied to a thin layer of black dye adhered to the skin, enabling controlled micro perforation without causing discomfort or tissue damage. The resulting openings facilitate gentle ISF extraction suitable for biosensing applications. The immunoelectrochemical device was subsequently fabricated through standard photolithographic processes on an oxidized silicon wafer under cleanroom conditions to ensure precision and reproducibility [28]. Gold microelectrode arrays functionalized with Dithiobis (succinimidyl propionate (DTSP) self-assembled monolayer (SAM) have been utilized to bind anti-cortisol (Mab) and to fabricate an ultrasensitive, disposable, electrochemical cortisol immunosensor. Ethanol amine (EA) was used to block nonspecific binding sites. The developed sensor has been demonstrated to possess the capability of detecting molecules within a linear range of 1 pM to 100 nM exhibiting a sensitivity of 0.325 M − 1. Another seminal work in this area is that of [29]. Nyquist plots derived from electrochemical impedance spectroscopy (EIS) were employed to examine variations in charge transfer resistance at the sensor–solution interface during each functionalization step, including DTSP self-assembled monolayer (SAM) formation, C-Mab antibody immobilization, and ethanolamine blocking. Additionally, the impedance data were analyzed to evaluate the changes in interfacial charge resistance in response to different cortisol concentrations and to determine the association constant for the cortisol–C-Mab interaction. The biosensor chips were fabricated on oxidized silicon wafers using standard photolithographic procedures to ensure precise patterning and reproducibility of the electrode structures [28]. A linear relationship was observed between the change in $R_{CT}$ values and the logarithm of cortisol concentrations for cortisol concentrations ranging from 1 pM to 1 μM, with a sensitivity of 1.165 kΩ/M and a correlation coefficient of 0.988. In a notable contribution, Yue Jing et al. [30] reported a microneedle-based electrochemical biosensor functionalized with cortisol-specific aptamers for minimally invasive monitoring in interstitial fluid (Figure 3). The device integrates a conductive gold-coated microneedle array that enhances electron transfer and provides a high surface area for aptamer immobilization. By exploiting an optimized surface chemistry for aptamer coupling and passivation, the system enables selective binding of cortisol directly within the skin interface. Electrochemical readout, performed via differential pulse voltammetry, revealed a broad linear detection range and sub-nanomolar sensitivity, demonstrating the feasibility of real-time hormonal assessment through ISF. This work exemplifies how microneedle-integrated aptasensors can bridge laboratory-grade electrochemical detection with wearable, pain-free platforms, offering a promising route toward continuous stress-biomarker tracking and personalized healthcare.

### 2.3. Saliva Analysis

The saliva has become an increasingly important biofluid for non-invasive cortisol monitoring, offering a convenient and stress-free alternative to blood or serum sampling. The simplicity, painlessness, and lack of requirement for trained personnel in saliva collection make it particularly well suited for repeated or ambulatory testing, with clear relevance for stress research, occupational health, and clinical diagnostics. Salivary cortisol has been shown to reflect the free, biologically active fraction of circulating hormone [31,32], with concentrations typically ranging from 1 to 7 ng/mL in the morning and decreasing to <1 ng/mL in the evening, closely following circadian rhythms. Notwithstanding the aforementioned advantages, salivary analysis is subject to analytical challenges. Cortisol concentrations in saliva are significantly lower than in blood, often in the low nanomolar to picomolar range, thus necessitating highly sensitive detection methods. Furthermore, saliva is a complex and variable matrix. Variables such as flow rate, circadian fluctuations, pH changes, and contamination from food or oral hygiene products can introduce noise and compromise reproducibility. These issues underscore the necessity for biosensors that can exhibit high sensitivity, selectivity, and robustness against matrix effects. In this context, electrochemical biosensors have attracted considerable attention. The potential for miniaturization, integration with portable electronics, and fast electrochemical readout renders them particularly suitable for point-of-care testing and wearable applications. In recent years, significant progress has been made in the field of antibody-based immunosensors, aptamer-based platforms, and molecularly imprinted polymers (MIPs). Many of these developments have already demonstrated feasibility for real-time salivary cortisol monitoring, thereby establishing the technological foundation for personalized stress assessment. Antibody-based immunosensors remain the most widely explored approach due to their high specificity. For instance, Dhull et al. [33] immobilized anti-cortisol antibodies onto a NiO thin film nanostructure deposited on an indium tin oxide (ITO) electrode. The incorporation of NiO nanostructures resulted in an augmentation of surface roughness and an enhancement of antibody loading, while the ITO ensured optimal conductivity. The device demonstrated an ultrasensitive detection limit of 0.32 pg/mL, with a linear dynamic range spanning from 1 pg/mL to 50 ng/mL. Its validation in real saliva samples substantiates both its sensitivity and clinical feasibility. In the field of antibody-based devices, exploration has been undertaken into the use of gold nanoparticles (AuNPs), carbon nanotubes (CNTs), and graphene oxide (GO) as immobilization and signal-amplification scaffolds. These studies have achieved detection limits in the low pg/mL range, representing significant advancements in the field. Aptamer-based sensors are gaining traction as synthetic, tunable, and regenerable recognition elements. Nguyen et al. [34] demonstrated an electrochemical aptamer-based (E-AB) platform for direct cortisol measurements in whole saliva without pre-treatment. The aptamer was modified with a redox probe (methylene blue) and immobilized on a gold electrode, thereby enabling signal transduction via square-wave voltammetry (SWV). This configuration demonstrated real-time reversible binding, regeneration after multiple cycles, and a dynamic range from approximately 100 picograms per milliliter to 1 microgram per milliliter, thus emphasizing its robustness in complex oral matrices. In other studies, the incorporation of graphene-modified electrodes and nanoporous gold structures has been demonstrated to enhance electron transfer and aptamer stability. Molecularly imprinted polymers (MIPs) have been shown to offer a low-cost, stable, and antibody-free alternative, through the fabrication of synthetic binding cavities that mimic the cortisol structure. A notable example is the “saliva-sensing dental floss” platform (Sharma et al. [35]), in which a cortisol-imprinted graphene electrode integrated into floss fibers enabled non-invasive detection in saliva absorbed along the floss. The device demonstrated an exceptional detection limit of 0.023 pg/mL, with a linear response spanning from approximately 0.01 pg/mL up to 1 µg/mL. This range encompasses both basal and stress-induced cortisol concentrations. The incorporation of graphene nanosheets ensured high conductivity and large surface area, while the MIP layer imparted stability against temperature and pH fluctuations. It has been reported by other MIP-based sensors that MXenes (Ti_3_C_2_T_x), metal–organic frameworks (MOFs) and conductive polymers (e.g., PEDOT:PSS) have been utilized as supporting matrices to further enhance selectivity and antifouling properties. Taking together, these approaches demonstrate that electrochemical biosensors are approaching clinical-grade performance, combining sub-picogram detection limits, wide dynamic ranges, and robustness in complex biofluids. Each recognition strategy antibody, aptamer, MIP offers distinct advantages in terms of sensitivity, stability, and cost, and their continued optimization will be central to the development of continuous, wearable cortisol monitoring technologies.

Whilst saliva provides several advantages for cortisol monitoring, it also presents notable limitations compared with sweat, particularly in wearable and continuous sensing contexts. Salivary cortisol concentrations are very low and highly variable, requiring ultra-sensitive detection and precise calibration. Measurements are easily affected by salivary flow rate, recent food or drink intake, oral hygiene products, and general mouth conditions, all of which introduce signal fluctuations. Moreover, the oral environment poses challenges such as adsorption, mixing delays, and limited diffusion zones, while contamination from enzymes or microorganisms can degrade the analyte. As an open system, saliva is also subject to concentration changes driven by hydration or evaporation, which compromise reproducibility. In contrast, sweat sampling occurs directly at the skin–sensor interface, minimizing contamination risks and enabling continuous collection. Thus, when designing reliable real-time cortisol biosensors, saliva’s intrinsic variability and instability make sweat a more suitable matrix for wearable applications.

### 2.4. Sweat Analysis

This paragraph expands upon a previously published framework on sweat biofluid analysis by the same authors [13]. The detection of cortisol in sweat has the potential to yield a novel methodology that is both immediate and minimally invasive. Cortisol concentration in sweat is found to be within the range of 8–141 ng/mL, exhibiting a strong correlation with the plasma concentration of cortisol [36]. Consequently, there is a necessity for a biosensor capable of ensuring reliability in detection within this range, thereby guaranteeing the linearity of the data obtainable. There are several practical constraints to consider for on-body operation, such as sampling variability, matrix effects, dependence on external redox probes, biofouling, and drift over time. In summary, although sweat sensing is feasible, it is not easy to plug-and-play. Robust devices pair compliant, antifouling electrodes with internal or ratiometric transduction and matrix calibration (pH/ionic strength) to minimize reliance on external probes and mitigate drift over multi-hour wear. Sweat has become an increasingly valuable source for hormonal monitoring, combining noninvasive sampling with the potential for real-time biochemical analysis. Its direct accessibility through the skin makes it particularly well-suited for integration into wearable devices aimed at assessing stress-related physiological changes. Although the concentration of cortisol in sweat is typically lower than in blood or saliva, advancements in sensor sensitivity and surface functionalization have made its reliable quantification increasingly feasible. Consequently, sweat-based cortisol detection is emerging as a promising approach for clinical and real-world stress assessment. In the following sections, we examine in detail the latest developments in wearable biosensors designed for this purpose, highlighting their technological innovations and analytical performance.

#### 2.4.1. Immunosensors

The present study explores the utilization of biosensors that employ antibodies for the detection of cortisol in sweat, a subject that has been extensively documented in extant literature. These antibodies exploit very strong interactions that are generated between the antibody and the antigen when they come into contact, thereby anchoring the target molecule on the surface of the working electrode. The most frequently employed electrochemical methods are DPV and EIS. Indeed, it is possible to quantify the concentration of the molecule in sweat by analyzing the decrease in current flow on the surface of the electrode, due to the loss of conductivity caused by the presence of an impedance, i.e., cortisol molecules. In this case, the concentration of cortisol will be inversely proportional to the current reduction inherent in the biochemical process. In this biosensor, it is imperative to achieve optimal immobilization of the biological component on the surface of the working electrode, thereby ensuring a substantial number of antibodies with a maximum quantity of active sites available. In this process, therefore, the directionality of the antibody molecules is equally important, that is, how they are arranged in space. A variety of methodologies can be employed to facilitate this process. This immobilization strategies relies on the use of bifunctional crosslinkers such as DTSP, which form strong thiol–gold and amide–protein bonds, thereby ensuring stable and oriented attachment of antibodies to the electrode surface. Such covalent coupling enhances the robustness of the sensing layer and preserves antibody activity for efficient antigen recognition.

**Figure 4:**
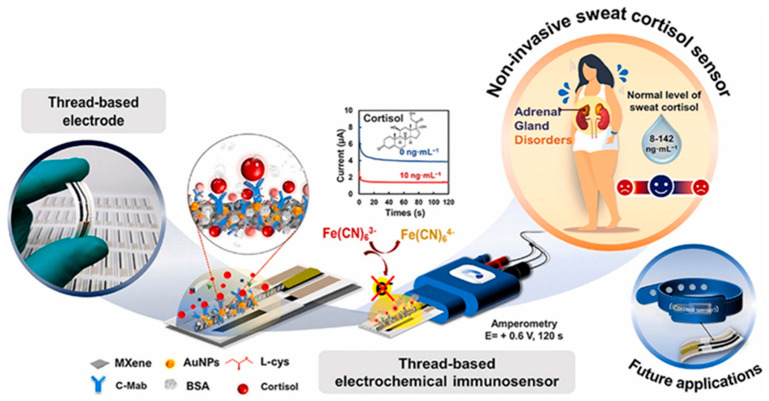
Illustration of an MXene-based platform designed for non-invasive measurement of cortisol in sweat. Reprinted with permission from [39]. Copyright 2025 Elsevier.

This review explores the diverse range of immunosensors, with the objective of fostering a comprehensive understanding of the variances and diversities that characterize this field of study. A significant proportion of the extant studies do not lend themselves to an analysis of cortisol concentrations in real time and continuously on an individual, using a wearable system. Indeed, the studies utilize external redox probes, such as ferricyanide, to ensure a consistent electrochemical response. However, this can only be achieved through the collection of sweat samples, which are then analyzed at a later point, thereby increasing the waiting period for patients. This is exemplified by the work of Tuteja et al. [37], in which samples are prepared at varying concentrations of cortisol. However, a redox probe is utilized to measure the electrical responses provided by the sensor via chronoamperometry. Some immunosensors use organometallic chemical components to increase the electrode surface area, which favors optimal immobilization of the biological component and improves transduction of the electronic signal. Examples of this include the work of Tian et al. [38] and Laochai et al. [39], who functionalize both working electrodes of the sensors with Mxene (Figure 4). MXene has unique physicochemical properties, including high charge carrier mobility, metallic conductivity, and favorable mechanical properties, and has a wide range of potential applications in sensors. In addition to these characteristics, biocompatibility is one of the most important features of MXene, making it a highly suitable matrix for producing advanced biosensing platforms. However, their application in the development of various sensors is inhibited by several drawbacks, such as low flexibility. Combining MXene with nanomaterials has been shown to be an effective way of making stable, highly conductive biosensors. Another example is provided by Tian et al. [40], who use metal–organic frameworks (MOFs). MOFs are a promising class of multifunctional materials characterized by porous structures, high specific surface areas and numerous catalytic active sites, making them highly suitable for use in immunological sensing. The precise arrangement of antibodies on MOF nanocrystals preserves their antibody–antigen-specific recognition properties while leveraging the beneficial properties of MOF materials. As previously mentioned in this article, the immobilization of antibodies is of the utmost importance. For this reason, cross-linkers such as DTSP are often employed. In their study, Upasham et al. [7] demonstrated a sweat-based electrochemical immunosensor for cortisol monitoring that highlights the role of molecular interface engineering in biosensor performance. The authors employed thiol–amide crosslinkers, such as 3,3′-dithiodipropionic acid di-N-hydroxysuccinimide ester (DTSP), to achieve stable and oriented immobilization of antibodies on gold electrodes. This approach ensured strong and reproducible binding, improving signal stability and overall sensitivity. Their work exemplifies how optimized surface chemistry, particularly the use of robust covalent coupling, can enhance selectivity and durability in wearable cortisol biosensors designed for noninvasive, continuous stress monitoring. Finally, an interesting study involved creating a biosensor that did not require batteries for power and could communicate wirelessly to study the obtained data. Cheng et al. [41] achieved satisfactory in situ cortisol detection results using a wearable, stretchable biosensor. This type of work undoubtedly opens the door to the real-time, continuous detection of cortisol, enabling the monitoring of variations in concentration in situ and in line with the circadian cycle.

#### 2.4.2. Molecularly Imprinted Polymers

**Figure 5:**
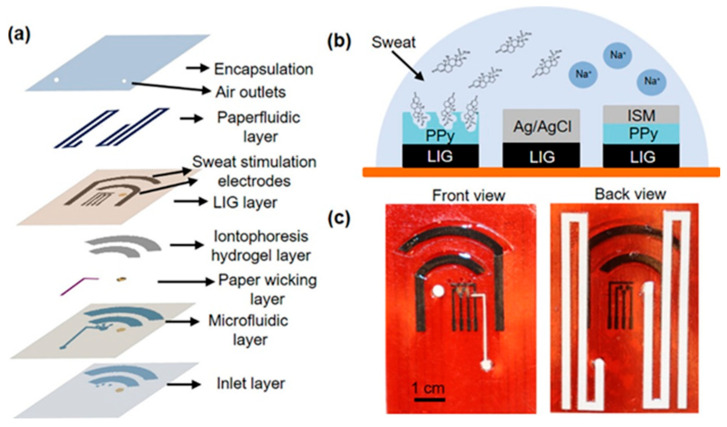
Wearable device layout. (**a**) Layered view highlighting structural components. (**b**) Schematic of the electrochemical sensor, comprising a laser-induced graphene (LIG) counter electrode, Ag/AgCl-coated LIG reference electrode, cortisol-specific MIP LIG working electrode, and a sodium ion-selective PPy/LIG electrode. (**c**) Optical photographs of the assembled patch, with the front view showing the skin-contact side. Reprinted with permission from [42]. Copyright 2025 Elsevier.

It is evident that another valid type of biosensor that can be used for the detection of cortisol in sweat is that which uses MIPs. Molecular imprinted polymers are synthesized directly on the surface of the working electrode via electropolymerizing, using a template to generate surface pockets specific to the molecule of interest. After the elimination of these template molecules through the process of over oxidation, the electrode surface is prepared to interact with the cortisol molecules present in sweat. It has been demonstrated that, by undertaking this course of action, the compound is hosted within the pockets of the material under investigation. This, in turn, results in a reduction in the conductivity of the electrode. Consequently, the concentration study of the analyte is conducted, once again, via impedance. An exemplar of such an application is furnished by Garg et al. [42], in which the detection of cortisol occurs via electrochemical impedance spectroscopy (Figure 5). Furthermore, multimodal electrochemical sensors with a sweat extraction module using iontophoresis and paper-based microfluidics are available. It is possible to quantify multiple parameters in parallel, including sweat volume, secretion rate, sodium, and cortisol concentration. Additionally, a microfluidics module has been incorporated. Although the present review does not focus on field-driven microfluidics, it is worth noting that recent studies have demonstrated how AC electric fields can actively enhance transport and assembly phenomena in soft colloidal and immunoassay systems. Polarization-selective dynamic coupling in rotating fields has been used to control electrorotation–orbital motion of twin colloids, while many-body electrohydrodynamic contact dynamics in AC dielectrophoresis have clarified hierarchical assembly pathways in soft binary suspensions. In parallel, numerical investigations of multiple-frequency AC electrothermal convection have shown that such flows can significantly intensify analyte transport and improve the efficiency of microfluidic immunoassays [43,44,45]. These AC electrokinetic and AC electrothermal strategies, although beyond the current scope centered on cortisol, could in the future be combined with wearable sweat patches to mitigate diffusion limitations and low sweat-rate conditions in microfluidic cortisol biosensors. A significant proportion of professional roles are not conducive to the implementation of wearable biosensors; indeed, they utilize external redox probes for the purpose of cortisol detection. Song et al. [46] sought to identify a solution regarding the utilization of external redox probes, integrating Prussian blue as a built-in redox probe. In this work, the molecularly imprinted polymer layer is produced through a two-step process involving the simultaneous electro-polymerization of Prussian Blue (PB) and pyrrole (Py) using cortisol as an imprinting template. This is followed by the elution of the cortisol template through the overoxidation of polypyrrole (PPy). The embedment of PB has the potential to serve as a redox current output mechanism in the absence of an additional labeling procedure and complex external probes, attributable to its abundant iron resources and high theoretical capacity. The sensor’s performance is then subjected to a chronoamperometric test, conducted in cortisol solution and artificial sweat, with the objective of evaluating its dependence on cortisol concentration, consistency and selectivity. It is possible to render MIPs extremely sensitive and rapid in detecting target molecules. As demonstrated by Mani and Anirudhan [47], allylated gold nanoparticle-incorporated carboxylated graphene oxide (allylated Au/GO-COOH) is utilized in the formation of a molecular cortisol imprinted polymer (Cor-MIP with Cor), thereby facilitating ultrasensitive and expeditious cortisol detection. The template Cor and the monomers methacrylic acid (MAA) and methyl methacrylate (MMA) were interconnected through hydrogen bonding. This facilitates the binding or rebinding of the Cor molecule in the polymer matrix, thereby enhancing its efficacy. The characterization of the functionalization and the measurement of the solutions with known cortisol concentration were achieved by employing cyclovoltammetry, EIS and DPV, respectively. Another type of application is given using mimic enzymes. In the work of Yeasmin et al. [48] the sensor employs an enzyme mimic on a substrate of EC-MIP molecularly imprinted electrocatalytic polymers. This innovative method allows for the resolution of the typical limitations found in the use of conventional sensors based on MIPs. The EC-MIP sensor contains cavities in which cortisol is retained and undergoes a reduction reaction of its ketone group to alcohol. This reaction is catalyzed by the presence of (CuPcTS-doped polypyrrole) copper phthalocyanine tetra sulfonate, thus generating an amperometric signal. In contrast to MIP sensors, EC-MIP sensors offer distinct advantages in terms of signal transduction, as they can produce signals directly through the oxidation or reduction in target molecules, thereby eliminating the necessity for an external redox probe. It is evident that the successful polymerization of MIPs is of pivotal significance for the effective functionality of the sensor. One of the primary concerns for sensors operating in complex biological fluids pertains to nonspecific adsorption, more commonly referred to as biofouling. This phenomenon has the potential to result in overwhelming signal interference and sensor malfunction [49,50,51,52]. The development of antifouling materials is imperative in order to mitigate nonspecific adsorption. In the present study, an antifouling MIP sensor based on sodium alginate-functionalized poly(3,4-ethylenedioxythiophene) (PEDOT/SA) for the detection of cortisol in real samples is presented by Wank et al. [53] Within the cortisol-imprinted hydrogel, cortisol molecules initially interacted with SA through hydrogen bonding and electrostatic attraction before being extracted from the imprinted hydrogel to form the MIP. The analytical performance evaluation is conducted using electrochemical impedance spectroscopy.

#### 2.4.3. Aptasensors

**Figure 6:**
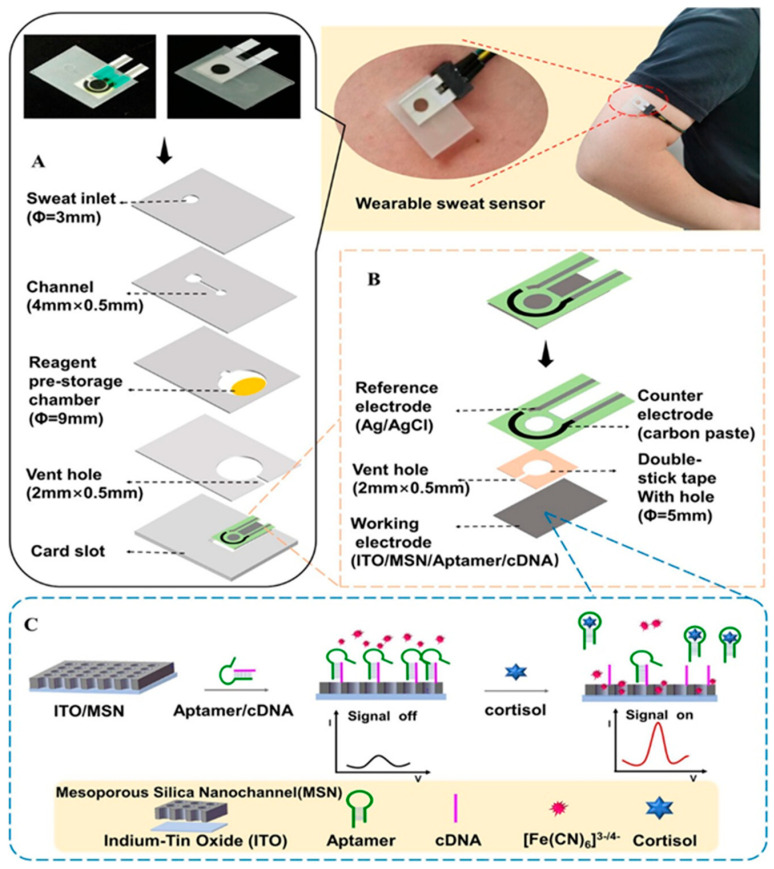
Scheme of the wearable sweat cortisol sensor with integrated reagents. (**A**) Device architecture in exploded form. (**B**) Detailed view of the three-electrode configuration. (**C**) Illustration of signal conversion at the working electrode. Reprinted with permission from [66]. Copyright © 2024 American Chemical Society.

A variety of probes can be utilized for the detection of cortisol, including aptamers. This type of biological compound is emerging as a leading candidate due to the advantages related to its use when compared to other compounds, such as antibodies and enzymes [54,55,56,57]. Indeed, the employment of aptamers has been demonstrated to reduce the necessity for reagents [58] and washing steps [59] whilst also facilitating sensor regeneration for real-time reversible measurements [60]. Aptamers are defined as single-stranded oligonucleotides that fold into specific architectures. The method of discovery employed was SELEX (Systematic Evolution of Ligands by Exponential Enrichment), a directed in vitro evolution technique. In this technique, large libraries of degenerate oligonucleotides are iteratively and alternately partitioned for target binding. Subsequent to this, the amplification of the functional sequences is achieved through the utilization of enzymes until the desired sequences are identified through the process of sequencing cloned individuals [61]. Notwithstanding the numerous advantages of their utilization, there are also disadvantages to be addressed. For instance, although aptasensors facilitate convenient biomarker detection, it is imperative to acknowledge that the sensitivity of the sensor is diminished by the high background current and low transduction efficiency [54]. This phenomenon occurs when aptamers bind to the target molecule, resulting in a change in conformation. This is due to the fact that the signaling molecule’s position in the unbound state is less well defined compared to its position in the bound state. In an attempt to surmount this issue, a considerable number of studies have proposed the utilization of a semi-rigid DNA strand within the aptameric structure, thereby ensuring that it is constrained to only adopt pre-established conformations [62]. A further salient limitation pertains to the fact that the affinity exhibited by aptamers for target molecules, in conjunction with the electrochemical properties of the redox probes employed to obtain the current signal, is contingent upon pH variations within the biological matrices, thereby hindering precise detection. The study by Singh et al. [63] is noteworthy for its attempt to resolve the aforementioned issues. The study proposes the creation of a wearable patch that is based on soft microfluidics and flexible electrodes. The patch is functionalized with a pseudoknot-assisted conformation switching aptamer. The purpose of this aptamer is to enable pH-calibrated, continuous, non-invasive cortisol monitoring in sweat. The sensor incorporates a flexible electrode, which is integrated with a microfluidic system. The electrode is functionalized via aptamers, which adopt two distinct conformations in response to variations in pH, as regulated by a pH sensor. The gold electrode is placed on a poly(dimethylsiloxane) (PDMS) base and consists of two working electrodes (one for pH and the other for cortisol measurement). The conformation-switched cortisol aptamer contains methylene blue (redox probe) at the distal end and short complementary regions interspaced with polythymine (T-12) spacers to form a pseudoknot structure. Upon binding of cortisol to the aptamer, a conformational change occurs, transitioning from a pseudoknot configuration to a loop structure. This adjustment positions the stem at the 3′-terminus, thereby enhancing the rate of electron transfer. This enhancement is attributed to the proximity of the redox probe to the electrode surface, which facilitates efficient electron transfer. This results in a change in current proportional to cortisol concentration. The sensor demonstrated the capacity to detect cortisol in artificial sweat solutions ranging from 1 pM to 1 μM, exhibiting an excellent LOD of 0.2 pM and satisfactory linearity (R^2^ = 0.98). Aptamers are typically not suitable for direct detection of primary sweat due to their sensitivity to both salinity and pH, which affects their affinity for targets [64,65]. In order to guarantee the right conditions, the authors Luan et al. [66] carry out saline and ionic additions to the sweat to “adjust” it and make the pH stable. The biosensor is composed of a microfluidic system, a three-electrode system and an interface for electrochemical signal conversion (Figure 6). The working electrode is composed of indium-tin-oxide (ITO), modified with silica micro-channels and aptamers. Once the binding with cortisol has occurred, the micro-channels open, modifying the electrochemical signal. The concentration study is carried out using differential pulse voltammetry. Another issue to consider when discussing wearable detection systems for biological matrices is the inevitable biofouling, which poses a significant obstacle [67,68]. This phenomenon gives rise to the generation of false signals and a diminution in the sensitivity of the biosensing system. Subsequently, Qiao et al. [69] developed a wearable antifouling biosensor. The sensor is composed of a polyaniline (PANI) hydrogel with hydrophilic polypeptides. The PANI three-dimensional cross-linked hydrogel network has the capacity to bind to a multitude of antifouling polypeptides, thereby contributing to the formation of a robust antifouling structure. This ensures the targeted detection of cortisol in a complex matrix such as sweat. The utilization of differential pulse voltammetry is twofold: firstly, to monitor the synthesis of PANI hydrogels, and secondly, to detect cortisol solutions. The detection process is followed in PBS and artificial sweat, covering a range from 10^−10^ g/mL to 10^−6^ g/mL, with a detection limit of 33 pg/mL. The sensor comprises three distinct layers: firstly, a layer of PANI hydrogel; secondly, a layer composed of the Sulfo- Succinimidyl- *trans*-4-(N-maleimidylmethyl)cyclohexane-1-carboxylate (Sulfo-SMCC) cross-linker; and finally, a layer consisting of polypeptides and aptamers for cortisol. In view of the necessity to develop a reliable and accurate sensor for the detection of biomarkers in sweat, it was hypothesized that the ratiometric electrochemical (EC) aptasensor could be an ideal candidate. Moreover, the employment of novel nanomaterials has been introduced to expedite the charge transfer and augment the catalytic activity, thereby facilitating the construction of a biosensor with exceptional specificity and sensitivity. An exemplar is provided by authors [70]. The construction of an aptasensor is achieved through the utilization of MOF organometallic compounds, which possess the capacity to interact with zirconium bonded to methylene blue. This latter component functions as an internal redox probe. Metal–organic frameworks (MOFs) represent a new generation of porous crystalline materials built from the coordination of metal nodes with organic linkers. Their architecture provides an exceptional degree of tunability, allowing control over pore geometry, chemical functionality, and overall stability. Owing to their large surface area, uniform porosity, and abundance of reactive sites, MOFs exhibit outstanding capacity for molecular adsorption and interaction. These properties have positioned them as highly versatile materials for diverse applications, ranging from catalysis and gas separation to sensing and biomedical technologies [71,72]. This renders them optimal candidates for utilization in sensor applications. Consequently, the complex is linked to the aptamers on the 5′ position via a phosphate group, with a strong Zr-O-P bond. The entire apparatus is situated on a gold surface that has been modified with multi-walled carbon nanotubes (MWCNTs), onto which the aptamers are anchored with cDNA. The utilization of an external iron-2-iron-3 redox probe is also a feature of the experimental setup. The ratio between the currents between the external and internal probe is calculated to obtain the concentration of cortisol that interacted with the aptamers. While the current generated by the external probe remains constant, the current generated by the internal probe decreases as cortisol binds to the sensor. The detection limit of the assay is 0.0046 nanomoles (nM), while its linear range extends from 0.1 nM to 1000 nM.

#### 2.4.4. Cross-Matrix Comparison of Cortisol Biosensor Performance

Some methodological considerations and background on sweat-based biosensing were previously discussed in our review on lactate monitoring [13]. Following a comprehensive analysis of the various sensors developed across distinct biological matrices, the main outcomes are summarized in Table 1, which systematically correlates the electrode materials, recognition strategies, linear range, and limit of detection (LOD) per biofluid. This comparative framework highlights how each configuration addresses the intrinsic chemical and physical constraints imposed by the target matrix, thereby outlining the key parameters that a cortisol biosensor must fulfill for analytical reliability and wearable applicability.

In blood-based systems, poly(3,4-ethylenedioxythiophene):polystyrene sulfonate (PEDOT:PSS) provides an efficient conductive interface capable of supporting electrochemical impedance spectroscopy (EIS) detection using minimal sample volumes. Its hydrophilicity and ionic mobility allow accurate drop-volume analysis without requiring any pretreatment step, which is advantageous for rapid, point-of-care assays.

In interstitial fluid (ISF), gold electrodes modified with 3,3′-dithiodipropionic acid di-N-hydroxysuccinimide ester (Au/DTSP) establish highly stable self-assembled monolayers (SAMs) that enable oriented antibody immobilization and low-picomolar immunodetection. Complementarily, gold nanoparticle-coated microneedle (MN/AuNP) platforms provide minimally invasive, transdermal access to ISF while amplifying electroactive surface area and signal-to-noise ratio. These architectures combine mechanical robustness with chemical specificity, two essential prerequisites for in vivo sensing.

Within saliva, both immunosensor and aptasensor approaches have been explored. Nickel oxide on indium tin oxide (NiO/ITO) achieves high catalytic activity and biocompatibility, enabling sub-picoliter detection limits. Gold-based aptasensors exploit the strong Au–S affinity for thiolated aptamer anchoring, ensuring selective cortisol binding even in the presence of complex salivary proteins. Moreover, graphene-based molecularly imprinted polymers (GPH-MIPs) demonstrate high recognition fidelity and ultra-low detection limits, benefiting from graphene’s π–π stacking and large specific surface area.

A wide array of electrode materials has been reported. Graphene-derived substrates, such as laser-induced graphene (LIG) and electro-reduced graphene oxide (e-RGO), provide extensive electroactive areas, high conductivity, and mechanical flexibility. Hybrid composites—such as MXene–multiwalled carbon nanotubes (MXene–MWCNTs) and L-cysteine/AuNP/MXene, further enhance immobilization efficiency and electron-transfer kinetics through synergistic effects between metallic and 2D conductive components.

Metal–organic frameworks (MOFs) support high bioreceptor loading capacities and enable ratiometric signal transduction, while Au/GO-COOH and Au/PB–MIP systems achieve label-free detection or incorporate internal redox probes to simplify signal readout. Conducting polymer matrices such as PEDOT/alginate (PEDOT/SA) and polyaniline (PANI) form soft, hydrated interfaces that mitigate biofouling and improve stability during prolonged on-skin operation. Additionally, indium tin oxide functionalized with mesoporous silica nanoparticles (ITO/MSN) maximizes the available surface area and probe density, which translates into higher sensitivity.

For molecularly imprinted polymer (MIP)-based transducers, electrochemically controlled architectures (EC-MIPs), such as CuPcTS-doped polypyrrole, enable direct transduction of the target’s redox activity, eliminating the dependence on external mediators. Similarly, Prussian Blue (PB)-embedded MIP films integrate the redox mediator within the recognition layer itself, improving operational stability and simplifying miniaturized circuit design. Both strategies represent critical advances toward robust, mediator-free, and calibration-light wearable platforms.

Taken together, these cross-matrix comparisons underscore how material engineering and recognition chemistry jointly dictate sensor performance in terms of sensitivity, selectivity, and long-term stability. As shown in Table 1, each matrix imposes unique physicochemical constraints—ionic strength, protein content, viscosity, and diffusion barriers—that must be balanced through material choice and transduction mechanism.

#### 2.4.5. Graphical Summary of Analytical Performance

**Figure 7:**
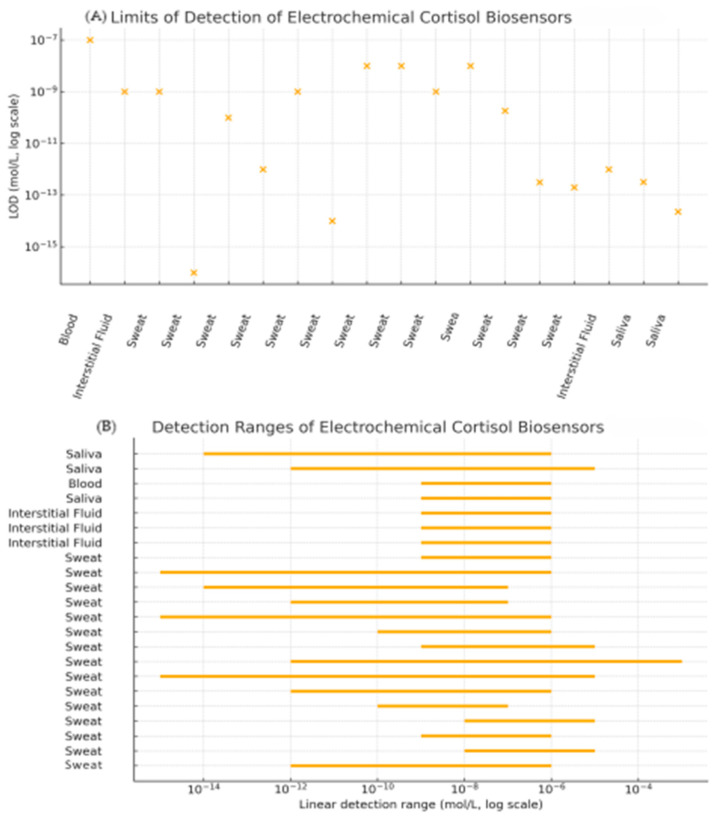
Graphical summary of electrochemical cortisol biosensor performance across biological matrices. (**A**) Limits of detection (LOD) for representative devices summarized in Table 1; left-to-right: [22], [30], [37], [38], [39], [40], [41], [44], [63], [66], [67], [7], [45], [50], [60], [29], [33], [35]; (**B**) Linear detection ranges for the same set of sensors plotted as horizontal bars on a logarithmic concentration axis; up-to-down: [35], [33], [22], [34], [23], [29], [30], [37], [38], [39], [40], [41], [42], [43], [44], [45], [50], [60], [63], [66], [67], [7].

In order to complement the numerical values listed in Table 1 and to facilitate a more intuitive comparison across devices and matrices, we extracted the reported limits of detection (LOD) and linear dynamic ranges of the most representative biosensors and summarized them in the comparative plots shown in Figure 7. Graph A displays the distribution of LODs on a logarithmic scale, grouped by biological matrix (blood, saliva, interstitial fluid, sweat). This visualization highlights how recent sweat-based platforms, particularly those using MXene–based immunosensors and advanced aptasensor or MIP architectures, have reached sub-picogram or sub-picomolar detection limits that are comparable to, or in some cases surpass, those reported in blood and saliva.

Graph B provides a graphical overview of the linear detection ranges of these devices in relation to the physiological cortisol concentrations in each biofluid. For each sensor, the reported linear range is plotted as a horizontal bar on a log-scale axis. This representation makes it possible to immediately identify which sensors fully span the relevant physiological window, which ones only cover basal or stress-elevated concentrations, and where gaps remain (e.g., devices with excellent sensitivity but a limited upper range). Taken together, these graphical summaries complement the tabulated values by providing a concise visual comparison of sensitivity and coverage across matrices, thereby improving the readability of the review and clarifying how current electrochemical platforms match the analytical requirements imposed by real physiological concentrations.

### 2.5. Comment on Clinical Applicability and Technological Limitations

Electrochemical wearable biosensors for sweat cortisol monitoring hold significant potential for clinical translation; however, their current role remains closer to exploratory and wellness applications than to routine medical diagnostics. From a clinical perspective, cortisol represents an attractive target because of its established role as a stress hormone, its circadian rhythmicity, and its use as a clinical biomarker in psychiatry, endocrinology, and occupational health. The capacity to monitor cortisol in a non-invasive manner through sweat eliminates the discomfort and logistical challenges associated with blood collection, while the potential for real-time, continuous tracking could facilitate novel insights into stress dynamics, fatigue, and recovery. In addition, the integration of cortisol sensors into wearable and mobile platforms, together with the possibility of combining cortisol readouts with other sweat analytes (e.g., lactate, electrolytes), further underscores their significance for personalized health management.

Despite this promise, there are significant technological challenges specific to cortisol that currently limit clinical translation. Cortisol is typically present in sweat at very low concentrations (5–250 ng/mL), often one to two orders of magnitude lower than in serum, and exhibits high inter- and intra-individual variability influenced by sweat rate, hydration status, and circadian phase. This necessitates sensors with ultrasensitive limits of detection and robust antifouling strategies to cope with matrix effects. Furthermore, the induction and regulation of sweat remain problematic: passive sweat may be inadequate for reliable cortisol quantification, whereas artificial induction methods (e.g., pilocarpine iontophoresis) add complexity and reduce wearability. Sensor instability over prolonged use is another limitation, particularly for antibody- and aptamer-based recognition layers, which can suffer from biofouling and signal drift. Although molecularly imprinted polymers (MIPs) offer greater intrinsic stability, they still require further optimization to achieve sufficient selectivity. Compounding these issues, a paucity of standardized calibration protocols and limited inter-study comparability complicate benchmarking and regulatory evaluation, and the correlation between sweat cortisol and serum free cortisol has not yet been fully validated across diverse populations, partly due to lag times in secretion and local skin transport mechanisms. Collectively, these factors indicate that while sweat-based cortisol biosensors hold clear promise for non-invasive, real-time stress monitoring, their immediate clinical utility is constrained by challenges in sensitivity, stability, and systemic correlation. Within this landscape, the choice of biorecognition strategy is critical. For clinical diagnostics, antibody immunosensors currently offer the clearest regulatory pathway and high specificity, but they require careful orientation chemistry and remain susceptible to drift and biofouling during prolonged wear. Aptamers enable reversible, reagentless monitoring with rapid regeneration and are readily integrated into electrochemical architectures; however, their affinity and transduction are sensitive to pH and ionic strength, and they may be prone to nuclease degradation and non-negligible background currents when densely packed at the interface, thereby motivating pH-calibrated or ratiometric designs. MIPs, by contrast, stand out for wearables because they are low-cost, solvent- and pH-tolerant, and regenerable; electrochemical MIPs and Prussian-Blue–embedded films provide built-in transduction that minimizes reliance on external mediators and can reduce drift. In this context, antibodies appear best suited to lab-grade validation and early clinical studies, aptamers to reversible continuous sensing when pH and ionic conditions are well controlled, and MIPs to durable, low-cost wellness devices operating in complex sweat, particularly when combined with antifouling hydrogels and compliant electrodes. Overall, aptamer- and MIP-based approaches should be regarded as complementary rather than mutually exclusive strategies. Aptamers provide high-affinity, sequence-tunable recognition and powerful opportunities for signal engineering, but their environmental sensitivity and potential instability must be carefully managed. MIPs, in turn, offer an antibody- and aptamer-free route to synthetic recognition layers with attractive thermal and chemical robustness and potentially lower cost, yet they still face important challenges related to biocompatibility, selective recognition of cortisol in the presence of structurally related steroids and other interferents, and reversible regeneration of binding sites under mild, on-body-compatible conditions. While molecularly imprinted polymers have often been portrayed as a “superior” alternative, in the specific context of wearable cortisol sensing they are more realistically viewed as a promising complementary technology with distinct strengths and limitations. Their robustness, synthetic tunability and compatibility with scalable fabrication clearly make them attractive for integration into flexible patches and textile platforms; however, issues of biocompatibility, selectivity under realistic sweat conditions, and long-term, in vivo regeneration and stability remain to be fully resolved before MIP-based cortisol wearables can be deployed in real-world settings. In order to bridge these gaps, advances in nanostructured transducers (e.g., graphene, MXenes, MOFs), antifouling and biocompatible coatings, and hybrid sensing platforms capable of contextualizing cortisol fluctuations with auxiliary physiological parameters (such as sweat rate, temperature and motion) will be required. Large-scale clinical trials will be essential to establish robust sweat–serum correlations and to satisfy regulatory requirements, thereby paving the way from proof-of-concept prototypes to clinically deployable stress monitoring tools that can operate reliably in everyday environments. Table 2 summarizes the practical trade-offs between different recognition elements (antibodies, aptamers, and MIPs)—including cost, durability, and ease of fabrication—in the context of specific application domains such as clinical diagnostics and wellness monitoring.

**Figure 8:**
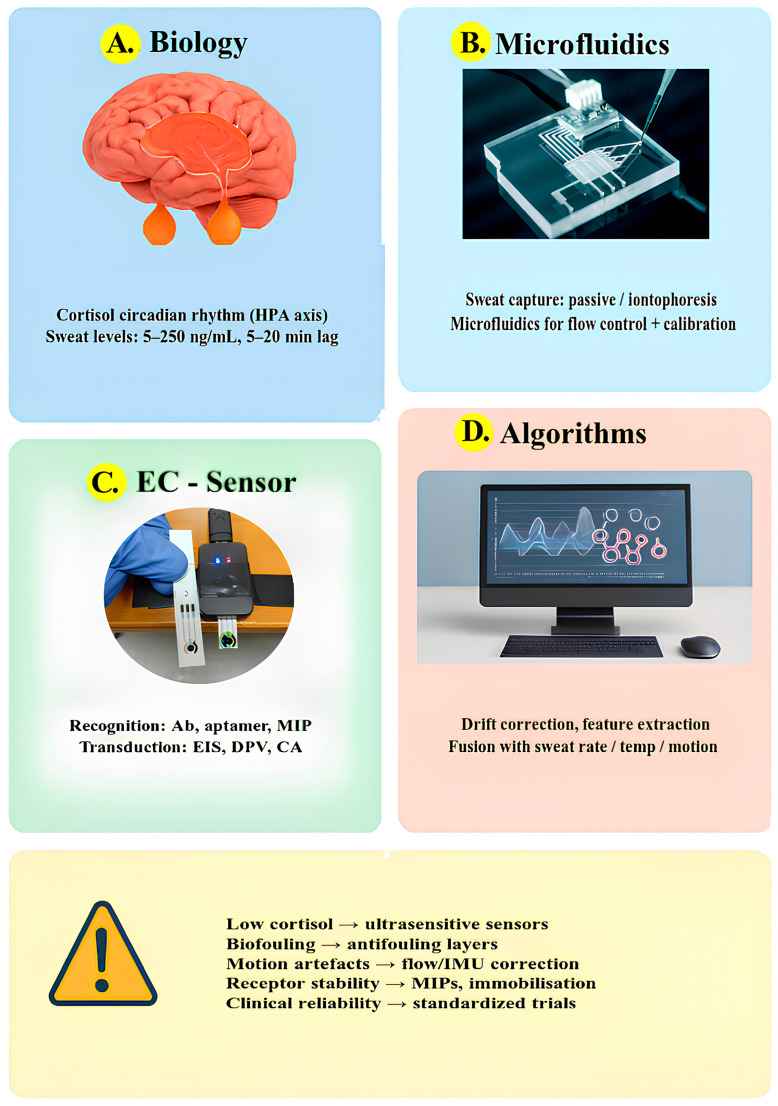
Clinical applicability and technological limitations of wearable electrochemical biosensors for sweat cortisol: (**A**) Biological context, (**B**) Microfluidic sampling, (**C**) Sensing strategies, (**D**) Data algorithms; key challenges and mitigations are summarized below.

Furthermore, Figure 8 provides an overview of the main aspects that determine both the clinical applicability and the current technological limitations of wearable electrochemical biosensors for sweat cortisol monitoring. The figure is organized into four sections, each illustrating a key component of this multidisciplinary system. Overall, the figure summarizes how biological, chemical, and engineering dimensions intersect in the design of wearable cortisol sensors, and how overcoming challenges such as low analyte levels, biofouling, and signal drift will be key to their future clinical translation.

## 3. Correlation Analysis Between Diverse Biological Matrices

As previously stated, blood serves as the primary biological matrix for the study of significant molecules present within the human body. The nominal value of cortisol in blood is characterized by a concentration range of approximately 20–250 ng/mL [19]. In order to detect the presence of this particular analyte in other matrices, it is imperative that there is a direct correlation with the concentration of the analyte that is usually present in blood.

In this respect, interstitial fluid (ISF) represents a particularly promising surrogate, as its biochemical composition closely mirrors that of plasma blood. Indeed, molecules and proteins have been observed to move towards ISF in a manner analogous to their movement from capillaries to cells. The continuous homeostatic processes present in the human body mean that these molecules have a direct correlation with the blood [25,26]. Consequently, the concentration of metabolites in the ISF is proportional to that present in the capillaries and is also based on the size of the molecule under examination [73,74]. Specifically for cortisol, its relatively small molecular weight (362.5 g/mol) and lipophilic nature facilitate diffusion across capillary and glandular epithelial membranes [75]. In comparison with blood, the hormone is present in the ISF with a minimal concentration of 12–34 ng/mL in the morning and 9–13 ng/mL in the evening [23].

Whilst saliva also offers significant advantages as a non-invasive matrix for cortisol monitoring, it presents several inherent disadvantages when compared with sweat, particularly in the context of wearable or continuous sensing. Salivary cortisol levels are relatively low and subject to rapid temporal fluctuations, requiring highly sensitive and stable detection systems. Measurements are strongly influenced by factors such as saliva secretion rate, recent food or drink intake, mouthwash residues, and oral hygiene practices, all of which can introduce bias or noise. Additionally, the oral environment is prone to adsorption effects, mixing delays, and the formation of “dead fluid zones” that limit analyte diffusion to the sensor surface, leading to slower equilibration and reduced accuracy. Further complications arise from contamination by enzymes, microbes, or particulates, which can degrade cortisol or interfere with detection chemistry. Because the mouth constitutes an open and highly variable environment, analyte concentrations fluctuate due to hydration, flow rate, and evaporation, factors that complicate calibration and reproducibility.

In contrast, sweat sampling circumvents many of these challenges. The biofluid is in direct contact with the sensing surface, minimizing sample handling and eliminating risks associated with ingestion or contamination. Sweat contains unbound cortisol, which is excreted from either the apocrine or eccrine glands. In normal circumstances, such as at rest or in healthy individuals, levels normally range between 5 and 50 ng/mL Cortisol concentrations can reach 200 or even 250 ng/mL in the presence of severe physical or emotional stress, extreme sporting activity, or in people with specific endocrine disorders [76]. A substantial body of research has demonstrated a strong correlation between the levels of free cortisol in saliva and blood [31,32]. As with salivary cortisol, there is interest in the use of sweat cortisol as a non-invasive biomarker for the monitoring of stress and circadian-related disorders, with a view to aiding the prevention of disease or the acceleration of recovery. As demonstrated in [77], cortisol secretion exhibits a diurnal pattern, and the concentrations observed have been shown to be comparable to those measured in saliva [36,78]. However, only the study of Torrente-Rodriguez [78] made a comparison between cortisol concentrations in sweat and those in blood. The authors postulate that circulating cortisol molecules are transported to and stored in eccrine and apocrine glands, secreted into the sweat, and ultimately excreted through a sweat pore to the epidermal surface [79]. It is hypothesized that cortisol levels present in sweat may be subject to circadian rhythm regulation by the internal clock and the light/dark cycle. The pilot study under consideration involved the monitoring of sweat cortisol variations over a period of six days in a healthy subject. Cortisol levels exhibit a distinct circadian rhythm, characterized by elevated concentrations in the early morning (AM) and markedly lower levels in the late afternoon or evening (PM). This diurnal pattern mirrors the physiological secretion cycle of circulating cortisol in blood. To investigate the relationship between sweat cortisol and systemic cortisol dynamics, samples of sweat, saliva, and serum were collected from four healthy individuals during the early morning and late evening. Consistent AM–PM variations were observed across all biological fluids, confirming that sweat cortisol closely follows the circadian fluctuations seen in serum and saliva. Statistical analysis revealed strong correlations between sweat and serum (r = 0.87) as well as between sweat and saliva (r = 0.78), underscoring the reliability of sweat as a noninvasive biomarker for monitoring hormonal rhythms. These findings suggest that sweat may serve as a valuable alternative matrix for tracking endocrine activity and stress-related physiological responses without the need for invasive sampling procedures.

When blood, saliva, interstitial fluid (ISF) and sweat are considered side by side, they all reflect cortisol dynamics but with distinct strengths and limitations. Saliva closely tracks free (biologically active) cortisol in blood and is only mildly affected by age, making it a robust non-invasive matrix for diurnal profiling. ISF, sampled via microneedles or hydrogels, also mirrors blood well with a small, modellable lag due to tissue diffusion. Sweat is more fragile: correlations with blood can be good during exercise or acute stress, but they depend heavily on sweat rate, collection method, and appropriate normalization for flow and sample age within the device. On top of this baseline, factors such as aging, autonomic or sweat disorders, metabolic and skin conditions, and environmental or behavioral influences (e.g., heat, humidity, physical activity, sampling time of day) can either strengthen or weaken cross-matrix agreement depending on whether devices measure and correct for them. In practice, calibrated saliva or ISF are preferable for clinical and circadian applications, whereas sweat becomes particularly attractive for real-time stress and exercise monitoring, provided that wearable patches incorporate robust microfluidics, flow normalization, and time-stamping.

Taken together, these findings indicate that, although saliva remains an important non-invasive fluid for cortisol assessment, sweat provides superior compatibility with continuous, wearable sensing platforms, owing to its accessibility, reduced contamination risk, and potential for real-time monitoring. Nevertheless, the biochemical complexity of sweat and its dependence on secretion dynamics necessitate careful calibration and validation against serum standards. As demonstrated in Table 3, a comparative analysis of the major biological matrices (blood, ISF, saliva, and sweat) illustrates the distinct cortisol concentration ranges.

As cortisol exhibits a pronounced diurnal rhythm, the majority of wearable and point-of-care studies utilize simple, model-agnostic summaries to contextualize signals, rather than considering the full chronobiology. The most common approach involves the adjustment of measurements for circadian phase by indexing each measurement to time since awakening (or fixed AM/PM windows) as a covariate. This is followed by the reporting of deviation from a personal baseline computed at the same time of day (e.g., the rolling median of the previous few days). Changes are then expressed as a percentage of a daily reference point (e.g., the AM peak or PM nadir) to facilitate cross-study comparison. In the context of sequential stress exposures, the practical implementation of tracking necessitates the collection of samples at the minute scale (ideally 1–2 min, with up to 5 min being acceptable) and the utilization of metrics that encompass kinetics, such as time to peak, half-recovery and area under the curve. It is imperative to acknowledge the existence of matrix-specific lags (ISF ~5–10 min, sweat ~10–20 min, depending on sweat rate). These lightweight options preserve scope while acknowledging key sources of variability and the dynamic activation-recovery cycling that is relevant to real-world use. Nevertheless, a standardized morning fasting measurement—analogous to clinical blood sampling—is widely regarded as essential to provide a stable reference point and to avoid confounding effects from circadian fluctuations.

## 4. Conclusions

In conclusion, the development of biosensors with sensitivity and linear detection range compatible with sweat cortisol concentrations appears to be a more viable prospect than ever before. Preliminary studies on the correlation between the presence of cortisol in blood and that in sweat also appear to permit the development of a biosensor capable of reliably evaluating its quantity. In future research, a more in-depth study of the physiological mechanism determining the presence of cortisol in sweat and further characterization of the correlation between sweat and circulating cortisol levels are required. The development of a biosensor for cortisol that can detect its concentrations in real time with reliability is therefore a significant and promising step towards achieving this goal.

The performance of wearable devices is contingent on a combination of material and transduction system properties, as well as the implementation of sophisticated algorithms. In practice, raw electrochemical signals (EIS/DPV) drift slowly over a period of minutes to hours due to changes in temperature, hydration, and the interface. In order to identify gradual trends and ignore transients, the implementation of adaptive baseline modeling is required. Simple low-order polynomials or autoregressive filters can already be effective, provided they are updated robustly to reject outliers, thereby keeping genuine event-related deviations visible while suppressing slow background wander.

Beyond hardware advances, the successful translation of wearable cortisol sensors will critically depend on appropriate time-series analysis and normalization strategies. Recent work on high-frequency endocrine profiling and circadian cortisol dynamics has shown that techniques such as rolling-median smoothing, intra-subject normalization to baseline profiles, and circadian phase–aligned averaging can robustly characterize overnight or 24 h cortisol patterns while reducing the impact of outliers and sampling noise [80]. In parallel, model-based analyses of the HPA axis and cortisol awakening response typically quantify dynamics in terms of phase, amplitude and area under the curve (AUC), providing a natural template for how dynamic metrics could be defined for wearable devices [81]. Furthermore, multimodal approaches that fuse wearable physiological signals with machine-learning or convolution-based models have already been used to estimate stress-related cortisol changes from heart rate, electrodermal activity and activity data, with validation against salivary cortisol measurements [82]. These studies collectively support the feasibility of applying more complex algorithms and normalization schemes to interpret continuous cortisol readouts.

There has been an increasing tendency to supplement classical signal-processing methods with artificial intelligence and machine learning approaches, which have been shown to facilitate more nuanced handling of complex signal patterns. The utilization of such tools has been demonstrated to enhance noise suppression, facilitate personalized calibration, and enable predictive modeling. Consequently, these tools contribute to an augmentation in the reliability and accuracy of wearable measurements when utilized under real-world conditions. Given the dynamic nature of sweat composition and flow, it is imperative that sensor output is interpreted in conjunction with concurrently measured matrix cues. The process of event detection should, in principle, be a simple and transparent one. The application of thresholds or change-point rules to baseline-normalized signals facilitates the interpretation of flags indicative of rises and recoveries. In circumstances where a greater degree of specificity is required, the incorporation of lightweight classifiers in conjunction with multimodal fusion (e.g., motion, skin temperature, or electrodermal activity) can facilitate the discernment of psychosocial stress responses from thermoregulatory sweating.

In order to be considered credible, it is essential that studies report on device-to-device and batch variability, cross-day stability and drift (%), interference and cross-reactivity (including related steroids), and methods of comparison to a reference assay (e.g., LC–MS/MS), using Bland–Altman/Deming statistics. To facilitate comparability between future wearable cortisol platforms, we recommend that, in addition to conventional analytical figures of merit (LOD, linear range, selectivity), a minimal set of dynamic metrics be reported for standardized protocols (e.g., stress tests, exercise, pharmacological challenges):

− Baseline-normalized cortisol-equivalent level at the start of the protocol (C_0_, accounting for time-of-day);
− Dynamic range or peak response (ΔC or fold-change relative to baseline);
− Time-to-peak (T_peak) after the defined stimulus;
− Half-recovery time (T_{1/2,rec}) from peak back toward baseline;
− Area under the curve over a predefined interval (AUC_0_−t), capturing overall exposure to elevated cortisol-equivalent levels;
− And short-term variability and signal drift over extended wear (e.g., coefficient of variation and 8–24 h drift).

When possible, these metrics should be calibrated and validated against sparse serum or salivary cortisol measurements, thereby linking wearable-derived indices to established clinical and chronobiological benchmarks. Analyses should be anchored to time-of-day baselines in order to handle diurnal effects. In addition, authors are required to state the intended use of the study (i.e., whether it is for wellness or diagnostic purposes), and to provide the corresponding evidence and information on regulatory expectations. When considered as a whole, these steps would result in the generation of cortisol time series that are both stable and comparable across days, thereby facilitating a more profound understanding of real activation–recovery dynamics. Despite the challenges that remain, the combination of standardized protocols, smarter algorithms, and multimodal sensing is gradually turning these challenges into opportunities, pushing wearable cortisol monitoring closer to reliable, everyday use in both clinical and wellness contexts.

Overall, this mini-review complements existing work by providing a systematic, cross-matrix comparison of electrochemical cortisol biosensors and by giving particular visibility to emerging sweat-based wearables and MIP-based recognition platforms. The quantitative juxtaposition of sensor performance and physiological cortisol ranges across blood, interstitial fluid, saliva and sweat is used to extract practical design guidelines for future wearable devices, including matrix-dependent requirements in terms of sensitivity, selectivity, antifouling strategies and system integration. In addition, the discussion of correlation studies, commercial and pre-commercial devices, and regulatory and standardization aspects bridges the gap between laboratory prototypes and clinically or societally relevant applications. Taken together, the review effectively synthesizes key advancements in cortisol biosensing and offers valuable perspectives on future directions, making it, we believe, a strong contribution to the field and to the journal’s readership.

With the rapid rise of wearable health technologies, the demand for reliable biosensors capable of tracking physiological stress markers such as cortisol is steadily increasing. Yet, despite intense scientific interest, no commercial device currently exists that can accurately and consistently measure cortisol in sweat. This absence reveals a crucial gap between laboratory research and real-world application, a space where innovation in materials, sensing strategies, and system integration could redefine the future of personalized stress monitoring. The “Corti wearable starter kit” has been identified as the sole sensor in this context (https://www.enlisense.com/ accessed on 26 November 2025). However, no information has been provided regarding the device, which is still indicated as being under development. Despite the existence of periodic announcements, no publicly available, peer-reviewed technical dossiers or clinical validation data for wearable cortisol products were found, highlighting a market information gap that currently precludes a fair comparison of prototypes or claims. The wearable cortisol sensing market is expanding, driven by growing awareness of stress and mental health, and by the broader shift toward preventative, personalized healthcare. Advances in electrochemical and optical biosensors, device miniaturization, wireless connectivity, and AI-based signal processing are enabling non-invasive, real-time monitoring through sweat and saliva. The field is also moving toward multimodal sensing, combining cortisol with physiological parameters such as heart rate, temperature, and skin conductance to improve stress assessment. However, despite this progress, commercially validated wearables remain rare. Table 4 shows the summary of prominent companies, prototypes, and approaches in cortisol sensing/wearables, and where they appear to be in development or commercialization. Most products are still at the prototype or promotional stage, and some rely on indirect physiological surrogates (e.g., electrodermal activity) rather than direct cortisol detection. Saliva-based spot-testing systems, such as cortiSense, are closer to market entry but still lack continuous monitoring capability.

The wearable cortisol biosensing sector remains at an early stage, with academic and startup prototypes showing strong innovation and sensitivity but limited long-term stability, calibration reliability, and large-scale validation. Further research and standardization are essential before clinical adoption. On-board data processing will move from simple baseline correction to hybrid pipelines that combine transparent signal processing with embedded AI, fusing cortisol with multimodal biosignals to extract individualized stress patterns while preserving interpretability. Standardized reporting, open datasets, and multicenter clinical trials will be key to validating sweat cortisol against gold-standard methods and clarifying its clinical utility. Commercially, early devices will focus on wellness and performance, while a later generation aims for medical-grade applications in endocrine and stress-related disorders, contingent on strong clinical evidence and careful attention to ethics, privacy, and data governance. In parallel, the increasing use of generative AI in scientific writing raises the risk of a proliferation of superficially sophisticated but experimentally under-validated papers, where complex analyses and claims are only weakly supported by primary data. Despite these challenges, the market outlook is highly positive: the European salivary cortisol wearable market exceeded USD 50 million in 2024 and is projected to reach €100–200 million by 2030, driven by AI integration, multimodal sensing, and technological advances. Once current barriers are overcome, continuous cortisol monitoring is expected to transition rapidly from prototype to widespread clinical and wellness applications.

## Figures and Tables

**Table 1:** Comparative analysis of electrochemical cortisol biosensors by various biofluids, electrode materials, and recognition modality. Material choices are grouped to highlight their role in electron-transfer kinetics, antifouling, and mechanical compatibility with wearables.

Working Electrodes	Matrix	Typology	Sensitivity	Linearity	Detection Limits	Ref.
PEDOT/PSS	Blood	Immunosensor	N/A	0.01–50 μg/dL	2.6 μg/dL	[22]
Au/DTSP	Interstitial Fluid	Immunosensor	2.296 kΩ/M	1 pM–100 nM	N/A	[23]
Au/DTSP	Interstitial Fluid	Immunosensor	1.165 kΩ/M	1 pM–1 μM	1 pM	[29]
MN/AuNPs	Interstitial Fluid	Aptasensor	N/A	1–1000 nM	0.22 nM	[30]
NiO/ITO	Saliva	Immunosensor	N/A	1 pM–10 µM	0.32 pM	[33]
Gold	Saliva	Aptasensor	N/A	0.1 nM–1 µM	N/A	[34]
GPH	Saliva	MIPs	N/A	0.01 pM–1 µM	0.023 pM	[35]
e-RGO	Sweat	Immunosensor	N/A	0.1–200 ng	0.1 ng/mL	[37]
MXene-MWCNTs	Sweat	Immunosensor	N/A	0.1 fg/mL–1 µg/mL	0.03 fg/mL	[38]
L-cys/AuNPs/MXene	Sweat	Immunosensor	N/A	5–180 ng/mL	0.54 ng/mL	[39]
MOF	Sweat	Immunosensor	N/A	1 pM–1 μM	0.26 pg/mL	[40]
Au/DTSP	Sweat	Immunosensor	N/A	1–200 ng/ml	1 ng/ml	[7]
AuNPs/SH-PEG-COOH	Sweat	Immunosensor	N/A	0–500 nM	7.47 nM	[41]
LIG	Sweat	MIPs	N/A	0.1 pM–1 μM	N/A	[42]
Au/PB	Sweat	MIPs	N/A	10^−9^ mol/L–10^−5^ mol/L	N/A	[46]
Au/GO-COOH	Sweat	MIPs	N/A	1 × 10^−3^ M–1 × 10^−14^ M	0.61 × 10^−14^ M	[47]
EC-MIP	Sweat	MIPs	N/A	0–1 μM	181 pM	[48]
PEDOT/SA	Sweat	MIPs	N/A	10^−12^–10^−8^ M	0.314 pM	[53]
Au	Sweat	Aptasensor	N/A	1 pM–1 μM	0.2 pM	[63]
ITO/MSN	Sweat	Aptasensor	N/A	10–5000 nM	8 nM	[66]
PANI	Sweat	Aptasensor	N/A	10^−10^–10^−6^ g/mL	33 pg/mL	[69]
MB@Zr-MOF	Sweat	Aptasensor	N/A	0.01–1000 nM	0.0046 nM	[70]

**Table 2:** Summary of the practical trade-offs between different biorecognition elements for sweat cortisol sensing.

Dimension	Antibodies	Aptamers	MIPs/EC-MIPs
Unit cost	$$–$$$ (biologic production, cold chain)	$$ (once selected; synthetic)	$ (commodity monomers)
Ease of fabrication	Moderate: oriented immobilization + blocking (e.g., DTSP, PEG)	Moderate: thiolated probes + redox labeling and passivation	High: electropolymerize + template removal; print-friendly
Operating stability	Sensitive to pH/temp/biofouling; drift over hours–days	Sensitive to ionic strength/pH; requires careful surface chemistry	Robust to pH/temperature/solvents; can be regenerated
Selectivity (steroids)	High, but cross-reactivity still possible	High if sequence is strong; some sequences weak for small steroids	Good but risk of class cross-reactivity (structural analogs)
Regeneration/reuse	Limited; harsh elution harms activity	Good (reversible, structure-switching E-AB)	Good; solvent/electrochemical regeneration
On-body compatibility	Proven in many prototypes; often needs external probes or MOFs/MXenes for signal	Works well with ratiometric/internal probes; needs pH/ionic calibration	Best fit for label-free or built-in redox (PB, EC-MIP) and antifouling hydrogels
Calibration burden	Medium–high (probe dependence, drift)	Medium (pH/ionic strength compensation)	Low–medium (mediator-free designs reduce drift)
Regulatory precedent	Strong (ELISA/CLIA heritage)	Growing but fewer cleared products	Limited for diagnostics; acceptable for wellness/PoC
Best fit	Lab-grade accuracy, clinical correlation studies	Reversible, continuous wearable sensing with smart calibration	Rugged, low-cost, continuous wellness wearables

**Table 3:** Comparative analysis of cortisol concentrations in biological matrices.

Matrix	Concentration
Blood	20–250 ng/ml
Saliva	1–7 ng/mL in the morning <1 in the evening
Interstitial Fluid	12–34 ng/mL in the morning 9–13 ng/mL in the evening
Sweat	5–250 ± 50 ng/ml

**Table 4:** Summary of prominent companies, prototypes, and approaches in cortisol sensing/wearables, and where they appear to be in development or commercialization.

Entity	Modality	2024–2025 Status	Notes
EnLiSense—Corti	Passive sweat; continuous cortisol and melatonin	Announced 2024; pre-market launch messaging	Claims real-time, continuous monitoring; independent performance data not yet public. (Business Wire)
Nutrix—cortiSense	Saliva, connected home test	CES 2025 award; at-home, non-continuous	~10 min readout; single-use test format; app integration. (ces.tech)
Xsensio—Lab-on-Skin™	On-skin biochemical sensing platform	Active platform/OEM development	Cortisol demonstrated in R&D; no public consumer SKU. (xsensio.com)
NOWATCH + Philips	Indirect (EDA) stress awareness	Commercial	Marketed for stress awareness; does not directly measure cortisol. (euronews)
Stressomic (academic)	Multiplex sweat (cortisol/EPI/NE)	Science Advances 2025	Signals direction toward multi-analyte products. (Science)

## Data Availability

As this is a review article, no data were generated.
